# The role of *NOTCH3* variants in Alzheimer's disease and subcortical vascular dementia in the Chinese population

**DOI:** 10.1111/cns.13647

**Published:** 2021-05-04

**Authors:** Lina Guo, Bin Jiao, Xinxin Liao, Xuewen Xiao, Weiwei Zhang, Zhenhua Yuan, Xixi Liu, Lu Zhou, Xin Wang, Yuan Zhu, Qijie Yang, Junling Wang, Beisha Tang, Lu Shen

**Affiliations:** ^1^ Department of Neurology Xiangya Hospital Central South University Changsha China; ^2^ National Clinical Research Center for Geriatric Disorders Central South University Changsha China; ^3^ Key Laboratory of Hunan Province in Neurodegenerative Disorders Central South University Changsha China; ^4^ Department of Geriatrics Neurology Xiangya Hospital Central South University Changsha China; ^5^ Department of Radiology Xiangya Hospital Central South University Changsha China; ^6^ Key Laboratory of Organ Injury, Aging and Regenerative Medicine of Hunan Province Changsha China

**Keywords:** Alzheimer's disease, CADASIL, *NOTCH3*, subcortical vascular dementia

## Abstract

**Aims:**

*NOTCH3* gene mutations predominantly cause cerebral autosomal dominant arteriopathy with subcortical infarcts and leukoencephalopathy, a common etiology of subcortical vascular dementia (SVaD). Besides, there may be a pathogenic link between *NOTCH3* variants and Alzheimer's disease (AD). We aimed to study the role of *NOTCH3* variants in AD and SVaD patients.

**Methods:**

We recruited 763 patients with dementia (667 AD and 96 SVaD) and 365 healthy controls from the Southern Han Chinese population. Targeted capture sequencing was performed on *NOTCH3* coding and adjacent intron regions to detect the pathogenic variants in AD and SVaD. The relationship between common or rare *NOTCH3* variants and AD was further analyzed using Plink1.9.

**Results:**

Five known pathogenic variants (p.R182C, p.C201S, p.R544C, p.R607C, and p.R1006C) and two novel likely pathogenic variants (p.C201F and p.C1061F) were detected in 16 SVaD patients. Additionally, no pathogenic or likely pathogenic variants were found in AD patients. *NOTCH3* was not associated with AD in either single‐variant association analysis or gene‐based association analysis.

**Conclusion:**

Our findings broaden the mutational spectrum of *NOTCH3* and validate the pathogenic role of *NOTCH3* mutations in SVaD, but do not support the notion that *NOTCH3* variation influences the risk of AD.

## Graphical abstract

This is the first systematic study of *NOTCH3* variants in a large Chinese cohort of Alzheimer's disease (AD) and subcortical vascular dementia (SVaD). The findings broaden the mutational spectrum of *NOTCH3* and validate the pathogenic role of *NOTCH3* mutations in SVaD, but do not support the notion that *NOTCH3* variation influences the risk of AD.

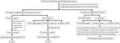

## 1 INTRODUCTION

Dementia is an acquired cognitive impairment syndrome, along with a decline in occupational and social functioning. As the most populous country in the world, China has the largest population with dementia.^1^ Alzheimer's disease (AD) is the most common dementia type, accounting for 2/3 of the dementia cases worldwide, and vascular dementia (VaD) is the second most common type.^2^, ^3^ VaD is mainly divided into multi‐infarct dementia, strategic infarct dementia, and subcortical VaD (SVaD) subtypes according to different pathogenesis. Compared with multi‐infarct dementia and strategic infarct dementia, SVaD often has an insidious onset, representing a more homogenous group.^4^ Both AD and SVaD present as cognitive decline in adults with insidious onset. Although the pathogenesis and pathology are different, AD and SVaD share many risk factors, including advancing age, and genetic and vascular risk factors. Vascular dysfunction also plays an important role in the pathogenesis of AD.^5^ Some pathology studies have shown that most patients with dementia had mixed pathologies, most commonly AD and vascular disease.^6^

*NOTCH3* encodes a transmembrane receptor mainly expressed in vascular smooth muscle cells (VSMC) and pericytes. The NOTCH3 protein is composed of an extracellular domain (ECD), a single transmembrane domain, and a noncovalently bound intracellular domain (ICD), and the ECD contains 34 tandem epidermal growth factor‐like repeat (EGFr) domains and three NOTCH Lin repeats. A pathogenic mutation in *NOTCH3* was found to cause cerebral autosomal dominant arteriopathy with subcortical infarcts and leukoencephalopathy (CADASIL), which is clinically characterized by migraine, recurrent subcortical strokes, and vascular cognitive decline or VaD in adults.^7^ CADASIL has been confirmed to be a common form of hereditary subcortical vascular cognitive impairment.^8^ Currently, more than 200 pathogenic mutations in the *NOTCH3* have been reported to cause CADASIL, and the majority are missense mutations distributing in exons 2–24, changing the number of cysteines in the EGFr domains, resulting in an odd number of cysteines in the ECD of the receptor, causing incorrect protein folding and aggregation.^9^, ^10^ Besides, an increasing number of *NOTCH3* missense mutations that did not affect the number of cysteines in EGFr domains were identified in suspected CADASIL patients, but the pathogenic role of these mutations was controversial because of undefined mechanisms.^9^, ^11^

Although CADASIL mainly manifests as vascular cognitive impairment, it may also present as an AD phenotype, with one Turkish patient clinically diagnosed with AD being found to carry a pathogenic mutation in the *NOTCH3*.^12^ Besides, *NOTCH3* was found to be associated with AD in a British and North American cohort,^13^ and another genetic study including participants of European ancestry showed that the *NOTCH3* rare coding mutations were significantly enriched in AD patients when compared with controls.^14^ However, there has not been any systemic study of *NOTCH3* variants in Chinese patients with AD and SVaD. It is necessary to investigate the frequency of pathogenic *NOTCH3* variants in Chinese patients with AD and SVaD and estimate the association between the *NOTCH3* variation and AD in a large Chinese cohort.

## 2 METHODS

### 2.1 Participants

Six hundred and sixty‐seven AD patients (male, 37.8%; mean age: 68.76 ± 11.35 years; 78 family AD and 589 sporadic cases) and 96 SVaD patients (male, 54.2%; mean age: 67.41 ± 11.10 years; 19 with family history and 77 sporadic cases) were recruited consecutively from Xiangya Hospital of Central South University, Changsha, Hunan, China (Table 1). All the AD patients met the diagnostic criteria of “probable AD dementia” recommended by the National Institute on Aging‐Alzheimer's Association (NIA‐AA) workgroups in 2011.^15^ All the SVaD patients met the SVaD diagnostic criteria established by Erkinjuntti et al^4^ This study also included 365 healthy elderly controls (male, 47.9%; mean age: 70.68 ± 5.35 years) from the physical examination center of Xiangya Hospital or communities of Changsha, all without obvious neurological disease. The Ethics Committee of Xiangya Hospital of Central South University approved the protocol of this study. Informed consent was signed by all the subjects according to the relevant regulations and guidelines.

**TABLE 1:** Clinic and demographic data of 667 AD patients, 96 SVaD patients, and 365 healthy elderly controls

	AD (*n* = 667)	SVaD (*n* = 96)	Control (*n* = 365)	*p*_1_	*p*_2_
Age (mean ± SD; median)	68.76 ± 11.35; 69.00	67.41 ± 11.10; 67.50	70.68 ± 5.35; 69.00	0.024^a^	0.011^a^
Age of onset (mean ± SD; median)	65.36 ± 10.99; 66.00	64.08 ± 10.74; 65.00			
Disease duration (mean ± SD; median)	3.41 ± 2.61; 3.00	3.32 ± 3.13; 2.00			
Gender (male/female)	252/415	52/44	175/190	0.002^b^	0.278^b^
Family history(+/−)	78/589	19/77			
MMSE (mean ± SD; median)	10.84 ± 7.26; 10.00	11.67 ± 7.80; 11.50	27.80 ± 1.51; 28.00	<0.001^a^	<0.001^a^
*APOE* allele frequency (ε2/ε3/ε4)	50/944/340	9/139/44	64/591/75	<0.001^b^	<0.001^b^
*APOE* genotype(ε4/non‐ε4)	284/383	36/60	72/293	<0.001^b^	<0.001^b^

Abbreviations: AD, Alzheimer's disease; *APOE*, apolipoprotein E; MMSE, mini‐mental State Examination; *p*_1_ represents *p* value between AD cases and controls; *p*_2_ represents *p* value between SVaD cases and controls; SVaD, subcortical vascular dementia.

^a^ *p* value was calculated by Mann–Whitney *U* test.

^b^ *p* value was calculated by chi‐squared test.

### 2.2 Library preparation and targeted sequencing

Genomic DNA was extracted from the peripheral blood leukocytes of each participant by phenol chloroform method. Capture probes were designed and customized for the coding regions and adjacent intronic regions of the *NOTCH3* gene (NM_000435). After genomic DNA was sheared by Bioruptor Pico Diagenode (Diagenode), the ends of the DNA fragments were repaired. The 5′ ends of the DNA fragments were phosphorylated, and a single adenine base was added to the 3′ ends by Exo(‐) Klenow. The adaptors were ligated to the ends with T4 DNA ligase, and the DNA fragments were amplified by polymerase chain reaction for 11 cycles. Targeted gene regions were captured with capture probes and isolated by bead purification. After the libraries were quantified with the Qubit 3.0 Fluorometer (Qubit dsDNA HS Assay Kit), sequencing was performed on the Illumina sequencing platform according to the manufacturer's protocols.

### 2.3 Bioinformatics processing and variants analysis

**FIGURE 1:**
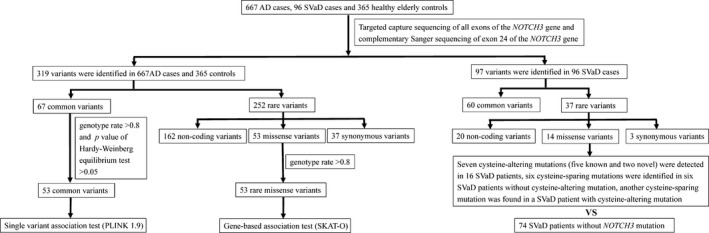
Flow chart of *NOTCH3* variants analysis in Chinese patients with AD and SVaD

The raw fastq sequences were aligned to the human genome v19 reference sequence with BWA.^16^ Variants were called with GATK^17^ and annotated with Annovar.^18^ The frequency of variants identified in this study was checked in public databases 1000 Genomes Project (1000Genomes, http://www.1000genomes.org/), the Exome Aggregation Consortium (ExAC, http://exac.broadinstitute.org), and the Genome Aggregation Database (gnomAD, http://gnomad.broadinstitute.org). The effect of missense variants was predicted by SIFT (http://sift.jcvi.org), PolyPhen2 (http://genetics.bwh.harvard.edu/pph2), Mutation Taster (http://www.mutationtaster.org), and Reve.^19^ All the pathogenic or likely pathogenic mutations in the *NOTCH3* gene were verified by Sanger sequencing. In the analysis of the association between dementia and *NOTCH3*, participants with a pathogenic or likely pathogenic mutation in *NOTCH3* were excluded. The flow chart of this study is shown in Figure 1. Due to the low coverage of exon 24, we performed Sanger sequencing on exon 24 for all subjects. Variants with minor allele frequency <0.01 in the gnomAD East Asian population, ExAC East Asian population, and 1000 Genomes database were defined as rare variants, and the remaining variants were common variants.

### 2.4 Statistical analysis

For common variants, Plink1.9 and logistic regression analysis (gender + age + *APOE* ε4 as covariates) were used to study the association between each variant and AD. For rare variants, sequence kernel association test‐optimal (SKAT‐O) was used to study the cumulative effect of *NOTCH3* rare missense variants on AD. Statistical analysis was performed using R (version3.3.0) and SPSS 20.0; and the Kolmogorov–Smirnov test (*n* > 50) and Shapiro‐Wilk test (*n* ≤ 50) were used to assess whether the measurement data are normally distributed. A *p* < 0.05 indicated a statistically significant difference.

## 3 RESULTS

### 3.1 Quality control and variants identification of *NOTCH3*

The average coverage rate of the target region of *NOTCH3* was 98.47%, and the average sequencing depth was 138.96×. Most of the encoding sequences have a coverage depth of at least 10×, except for parts of exons 1, 24, and 33. After filtering out variants of low quality (coverage < 10× or variants supporting depth accounts for the total depth <25%), 344 variants were identified by targeted sequencing (Table S1).

As exon 24 of *NOTCH3* is a hot spot region of pathogenic mutations, we performed Sanger sequencing of this exon in each sample to avoid false negatives. Furthermore, two rare variants were found in ten subjects by complementary Sanger sequencing, including p.P1354L in an AD patient and p.G1347R in nine subjects (seven AD, one SVaD, and one control). In the end, a total of 345 variants were identified by the targeted and Sanger sequencing.

### 3.2 Phenotypes of the patients with a pathogenic or likely pathogenic mutation in *NOTCH3*

Five known CADASIL pathogenic mutations in the *NOTCH3* gene (p.R182C, p.C201S, p.R544C, p.R607C, and p.R1006C) were identified in 14 unrelated SVaD patients (Table 2). Additionally, two novel likely pathogenic mutations (p.C201F and p.C1061F) were found in two SVaD patients, respectively. The two novel mutations were absent from 365 healthy control individuals and public population databases. Multiple online software predicted that both of the two novel mutations generate a deleterious effect (Table 2), and they should be classified as likely pathogenic variants according to the American College of Medical Genetics and Genomics (ACMG) standards and guidelines in 2015.^20^

**TABLE 2:** Seven pathogenic or likely pathogenic mutations in *NOTCH3* identified in 16 SVaD patients

Patient no.	Nucleotide change	Amino acid change	Exon	EGFr	SIFT	Polyphen‐2	Mutation taster	ReVe	Previously reported in CADASIL
1	c.544C>T	p.R182C	4	4	Damaging	Probably damaging	Disease causing	Damaging	Yes
2	c.602G>C	p.C201S	4	5	Damaging	Probably damaging	Disease causing	Damaging	Yes
**3**	**c.602G>T**	**p.C201F**	**4**	**5**	**Damaging**	**Probably damaging**	**Disease causing**	**Damaging**	**No**
4, 5, 6, 7, 8, 9, 10	c.1630C>T	p.R544C	11	13/14	Tolerable	Probably damaging	Disease causing	Damaging	Yes
11, 12, 13, 14	c.1819C>T	p.R607C	11	15	Tolerable	Probably damaging	Disease causing	Damaging	Yes
15	c.3016C>T	p.R1006C	19	26	Damaging	Probably damaging	Disease causing	Damaging	Yes
**16**	**c.3182G>T**	**p.C1061F**	**20**	**27**	**Damaging**	**Probably damaging**	**Disease causing**	**Damaging**	**No**

Note

Novel variants are in bold.

Abbreviations: CADASIL, cerebral autosomal dominant arteriopathy with subcortical infarcts and leukoencephalopathy; EGFr, Epidermal growth factor repeat; SVaD, subcortical vascular dementia.

**FIGURE 2:**
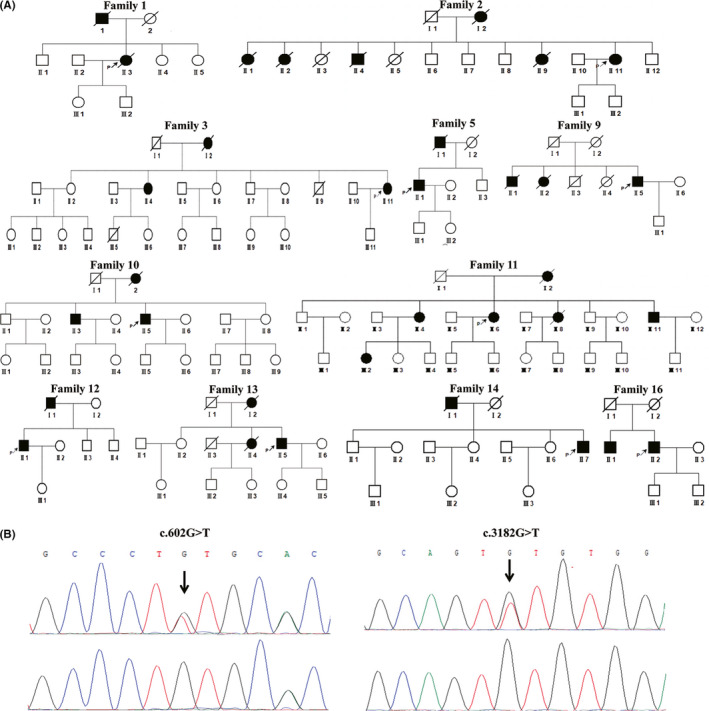
Family pedigrees and novel mutations. (A) Pedigree charts of 11 SVaD patients with a pathogenic or likely pathogenic mutation and positive family history. Squares indicate males, circles indicate females, black symbols indicate affected individuals, and arrows indicate the probands. (B) Sanger sequencing chromatograms of the two novel likely pathogenic variants

The clinical and imaging data of 16 unrelated patients (one males and six females) with a pathogenic or likely pathogenic mutation are summarized in Table 3. The age of onset ranged from 25 to 70 years, and the most common initial symptom was cognitive impairment (11/16), followed by stroke (3/16), and migraine (2/16). During the disease, common symptoms included cognitive decline (16/16), psychological and behavioral abnormalities (9/16), stroke (6/16), incontinence (6/16), gait disturbance (5/16), and migraine (4/16). Brian magnetic resonance imaging (MRI) showed significant white matter hyperintensities (WMH) (Fazekas scale ≥2) in 100% patients, 35.7% extended to the temporal pole, and 85.7% extended to the external capsule. Six patients underwent skin biopsy, and 100% were tested positive for characteristic granular osmiophilic material (GOM) deposits with electron microscopy. Eleven patients had a family history of cognitive impairment, and the family pedigrees are shown in Figure 2A. Sanger Sequencing chromatograms of the two novel likely pathogenic variants are shown in Figure 2B.

**TABLE 3:** Clinical and imaging characteristics of 16 SVaD patients with a pathogenic or likely pathogenic mutation in the *NOTCH3* gene

Patient no.	Sex/age/age at onset	Family history	Initial symptoms	Other symptoms	Fazekas scale		WMH		Small infarcts	GOM	APOE	MMSE
					Periventricular white matter	Deep white matter	Temporal pole	External capsule				
1	F/52/50	+	Stroke	Cognitive impairment, gait disturbance, and headache	3	3	−	+	+	—	ε2/ε3	6
2	F/59/57	+	Stroke	Cognitive impairment	3	3	+	+	+	+	ε3/ε3	12
3	F/47/46	+	Cognitive impairment	Stroke, psychological and behavioral abnormalities, gait disturbance, aconuresis	3	3	−	+	+	+	ε3/ε3	3
4	M/62/61	−	Cognitive impairment	Personality change, aconuresis, and bradykinesia	3	3	−	+	+	+	ε3/ε3	18
5	M/69/67	+	Cognitive impairment	Psychological and behavioral abnormalities, and involuntary movement	2	3	−	+	+	—	ε3/ε3	12
6	M/63/55	−	Cognitive impairment	Personality changes	3	3	−	+	+	—	ε3/ε4	24
7	F/74/67	−	Cognitive impairment	Stroke, psychological and behavioral abnormalities, aconuresis	—	—	—	—	—	—	ε3/ε3	0
8	F/76/70	−	Cognitive impairment	Psychological and behavioral abnormalities	—	—	—	—	—	—	ε3/ε3	3
9	M/71/70	+	Cognitive impairment	Psychological and behavioral abnormalities	2	2	−	−	+	—	ε3/ε3	16
10	M/68/66	+	Cognitive impairment	Migraine, psychological and behavioral abnormalities, gait disturbance, and encopresis	3	3	−	+	+	—	ε3/ε3	6
11	F/51/25	+	Migraine	Cognitive impairment, psychological and behavioral abnormalities	3	3	+	+	+	—	ε3/ε3	15
12	M/60/58	+	Stroke	Cognitive impairment	2	3	−	−	+	—	ε3/ε3	24
13	M/61/58	+	Migraine	Cognitive impairment	2	2	+	+	+	+	ε3/ε4	23
14	M/54/50	+	Cognitive impairment	Gait disturbance, psychological and behavioral abnormalities, aconuresis, and encopresis	3	3	+	+	+	+	ε3/ε3	14
15	M/46/41	−	Cognitive impairment	Stroke, psychological and behavioral abnormalities, and encopresis	3	2	−	+	+	—	ε3/ε3	13
16	M/70/69	+	Cognitive impairment	Gait disturbance, dizziness, and slurred speech	3	3	+	+	+	+	ε3/ε3	26

Abbreviations: *APOE*, Apolipoprotein E; F, Female; GOM, granular osmiophilic material; M, Male; MMSE, mini‐mental State Examination; SVaD, subcortical vascular dementia; WMH, white matter hyperintensities.

**FIGURE 3:**
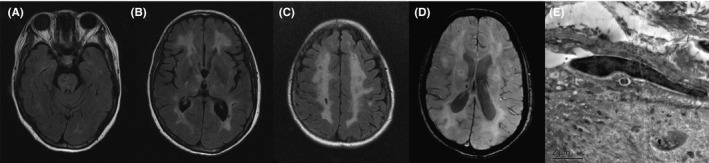
Brain MRI and electron microscopy pictures of the patient with the novel variant c.602G>T, p.C201F. Brain MRI of the patient at her 47 showed diffuse WMH on FLAIR(A‐C) and a few microbleeds on SWI(D). Skin biopsy of the patient showed GOM in the plasma membrane of a VSMC, ×7000 (E)

The c.602G>T, p.C201F variant in *NOTCH3* was detected in a 47‐year‐old female (patient 3, family 3), who began with psychological and behavioral abnormalities and cognitive decline at age 46. Then, her condition deteriorated quickly within 1 year. On admission, she had difficulty in communicating with others and controlling her bladder, and taking care of herself. Her performance on the mini‐mental state examination (MMSE) was severely impaired (3/30). Brain MRI demonstrated multiple lacunar lesions in the bilateral frontal‐parietal lobes and diffuse WMH in T2WI (Figure 3A–C), and susceptibility‐weighted imaging (SWI) showed cerebral microbleeds in the cortex, thalamus, cerebellum, and brainstem (Figure 3D). Her elder sister and deceased mother presented similar symptoms after the age of 60. *NOTCH3* mutation testing showed that the novel mutation was present in the affected elder sister (II:4) but was absent in her healthy eldest sister (II:2). Moreover, GOM was observed in the vascular wall of the proband's skin using an electronic microscope (Figure 3E).

**FIGURE 4:**
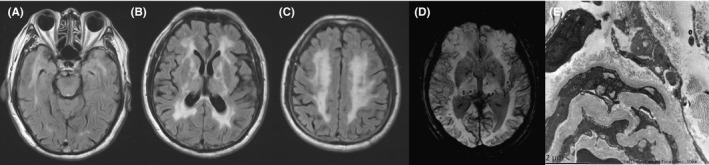
Brain MRI and electron microscopy pictures of the patient with the novel variant c.3182G>T, p.C1061F. His brain MRI at 70 showed diffuse WMH on FLAIR extending to the temporal pole and external capsule (A–C) and a few microbleeds on SWI (D). Skin biopsy of the patient showed GOM deposition at the cell surface of VSMCs, ×10,000 (E)

Another novel variant was detected in a 70‐year‐old male (patient 16, family 16), who experienced memory decline, slurred speech, and unstable walking gradually within 1 year. He had a history of hypertension for 2 years, and no other medical history was reported except cataract. Neurological examination showed inarticulate sounds, cognitive impairment, and a positive Romberg sign. The assessment of MMSE (26/30) and Montreal Cognitive Assessment (MoCA, 17/30) confirmed mild impairment in multiple domains. Brain MRI revealed diffuse involvement of WMH in the bilateral temporal lobe, periventricular white matter, and semioval center (Figure 4A–C). Furthermore, numerous microbleeds distributed in the frontal lobe, thalamus, pons, and right cerebellum were seen on SWI (Figure 4D). Genetic testing identified a novel characteristic CADASIL causing mutation (c.3182G>T, p.C1061F) in *NOTCH3*. Skin biopsy showed thickened walls of the small blood vessels and electron microscopic GOM deposits at the surface of VSMC (Figure 4E). His elder brother reported similar clinical and imaging characteristics but refused to further genetic evaluation and skin biopsies. Together with the above information, the diagnosis of CADASIL was confirmed.

### 3.3 Phenotypes of participants with a cysteine‐sparing missense mutation in EGFr domains of *NOTCH3*

Although most of the pathogenic mutations were located in the 34 EGFr domains and caused CADASIL by affecting the number of cysteines in EGFr domains, a few cysteine‐sparing mutations in EGFr domains were also identified in suspected CADASIL patients. As shown in Table 4, 33 cysteine‐sparing *NOTCH3* missense variants encoding for EGFr domains were found in our cohort, and all were rare variants except for p.A1020P and p.R1175W. It is noteworthy that six cysteine‐sparing variants (p.R75Q, p.P167S, p.V237M, p.A1020P, p.R1100H, and p.G1347R) previously reported associated with CADASIL were identified in our healthy controls. Moreover, five rare variants were also detected in 25 AD patients, but none differed significantly between AD patients and healthy controls (*p* > 0.05). Among 11 AD patients who carried the mutation p.G1347R, five met AD with dementia diagnostic criteria recommended by NIA‐AA in 2018,^21^ with CSF Aβ42 decreased or PIB‐PET positive and t‐tau or p‐181‐tau increased. One AD carrier diagnosed by biomarkers with moderate white matter lesions and numerous microbleeds (Figure S1A–D) underwent a skin biopsy, but no GOM was observed in the subcutaneous arterioles by electron microscopy (Figure S1E).

**TABLE 4:** Clinical diagnosis of subjects with a cysteine‐sparing missense variant in *NOTCH3* EGFr domains

Nucleotide change	Amino acid change	Exon	SVaD (*n* = 96)	AD (*n* = 667)	Control *(*n = 365)	Variant previously reported in CADASIL
c.4061C>T	p.P1354L	24	0	1 Het	0	No
**c.4039G>C**	**p.G1347R**	**24**	**1 Het**	**11 Het**	**1 Het**	**Yes** ^22^
c.3946C>T	p.P1316S	24	0	1 Het	0	No
c.3560G>A	p.G1187D	22	0	0	1 Het	No
c.3524G>A	p.R1175Q	22	0	0	1 Het	No
c.3523C>T	p.R1175W	22	0	8 Het	4 Het	No
c.3455C>T	p.T1152M	21	0	1 Het	0	No
**c.3299G>A**	**p.R1100H**	**20**	**1 Het**	**1 Het**	**1 Het**	**Yes** ^23^
c.3260C>T	p.P1087L	20	0	1 Het	0	No
**c.3058G>C**	**p.A1020P**	**19**	**0**	**0**	**1 Het**	**Yes** ^24^
c.2983C>T	p.P995S	18	0	1 Het	0	No
c.2906G>A	p.R969Q	18	0	0	1 Het	No
c.2234C>T	p.A745V	14	0	1 Het	0	No
c.1778A>G	p.H593R	11	0	1 Het	0	No
c.1690G>A	p.A564T	11	1 Het	0	0	No
c.1673G>A	p.R558H	11	0	0	1 Het	No
c.1508C>T	p.T503M	10	0	1 Het	0	No
c.1490C>T	p.S497L	9	0	1 Het	1 Het	No
c.1453A>G	p.K485E	9	0	1 Het	0	No
c.1265G>T	p.G422V	8	0	1 Het	0	No
c.1186T>G	p.S396A	7	0	1 Het	0	No
**c.709G>A**	**p.V237M**	**5**	**0**	**5 Het**	**2 Het**	**Yes** ^25^
c.590C>T	p.P197L	4	0	1 Het	0	No
c.515G>A	p.G172D	4	0	1 Het	1 Het	No
c.506G>A	p.R169H	4	0	0	1 Het	No
**c.499C>T**	**p.P167S**	**4**	**0**	**7 Het**	**4 Het**	**Yes** ^26^
c.482A>G	p.E161G	4	0	0	1 Het	No
c.415G>A	p.D139N	4	0	1 Het	0	No
c.391G>C	p.G131R	4	0	0	1 Het	No
c.391G>A	p.G131S	4	1 Het	0	0	No
c.373A>C	p.S125R	4	0	1 Het	0	No
c.269G>A	p.R90H	3	0	2 Het	0	No
**c.224G>A**	**p.R75Q**	**3**	**0**	**1 Het**	**1 Het**	**Yes** ^27^

Note

Variants previously reported in CADASIL are in bold.

Abbreviations: AD, Alzheimer's disease; Het, heterozygote; SVaD, subcortical vascular dementia.

### 3.4 Clinical features comparison between SVaD patients with and without *NOTCH3* mutations

After comparing the clinical features of SVaD patients with and without *NOTCH3* mutations, we found that those with *NOTCH3* mutations were younger (63.27 ± 9.28 years vs 68.64 ± 11.35 years, *p* = 0.046), presented an earlier onset age (60.05 ± 8.86 years vs 65.28 ± 11.01 years, *p* = 0.044), and a more frequent family history of cognitive impairment (50.0% vs 10.8%, *p* < 0.001) than the patients without *NOTCH3* mutations (Table 5). Moreover, SVaD patients with *NOTCH3* mutations tended to have more external capsule and temporal pole involvement than SVaD patients without *NOTCH3* though without significance (*p* = 0.059 and *p* = 0.076, respectively). No significant differences were observed between the two groups in the other clinical features analyzed, which included disease duration, gender distribution, MMSE scores, frequency of the *APOE* allele, stroke, and vascular risk factors.

**TABLE 5:** Comparisons of clinical features between SVaD patients with and without *NOTCH3* mutation

	Total (*n* = 96)	*NOTCH3* mutation (+) (*n* = 22)	*NOTCH3* mutation (−) (*n* = 74)	*p*
Age (mean ± SD; median)	67.41 ± 11.10; 67.50	63.27 ± 9.28; 64.00	68.64 ± 11.35; 69.00	0.046^c^
Age of onset (mean ± SD; median)	64.08 ± 10.74; 65.00	60.05 ± 8.86; 62.00	65.28 ± 11.01; 66.00	0.044^c^
Disease duration (mean ± SD; median)	3.32 ± 3.13; 2.00	3.23 ± 2.14; 2.00	3.35 ± 3.38; 2.50	0.621^d^
Gender (male/female)	52/44	13/9	39/35	0.598^e^
Family history(+/−)	19/77	11/11	8/66	<0.001^f^
MMSE (mean ± SD; median)	11.67 ± 7.80; 11.50	13.23 ± 8.75; 13.50	11.20 ± 7.50; 11.00	0.287^c^
*APOE* allele frequency (ε2/ε3/ε4)	9/139/44	1/36/7	8/103/37	0.268^e^
*APOE* genotype(ε4/non‐ε4)	36/60	6/16	30/44	0.259^e^
Vascular risk factors^a^	50/46	9/13	41/33	0.232^e^
Stroke (+/−)	40/56	9/13	31/43	0.935^e^
WMH in MRI^b^				
Fazekas scores (2/3)	20/41	4/13	16/28	0.338^e^
External capsule (+/−)	42/19	15/2	28/16	0.059^e^
Temporal pole (+/−)	12/49	6/11	6/38	0.076^f^

Abbreviations: APOE, apolipoprotein E; MMSE, Mini‐mental State Examination; MRI, magnetic resonance imaging; *p* represents p value between SVaD with *NOTCH3* mutations and SVaD without *NOTCH3* mutations; SVaD, subcortical vascular dementia; WMH, white matter hyperintensities.

^a^ Including smoking, hypertension, and diabetes.

^b^ Only including patients with complete MRI available.

^c^ *p* value was calculated by independent‐samples *t* test.

^d^ *p* value was calculated by Mann‐Whitney U test.

^e^ *p* value was calculated by chi‐square test.

^f^ *p* value was calculated by Fisher's exact test.

### 3.5 Cysteine‐sparing *NOTCH3* mutations in SVaD patients without cysteine‐altering *NOTCH3* variant

Among the 80 SVaD patients without the cysteine‐altering *NOTCH3* variant, six carried a cysteine‐sparing *NOTCH3* mutation. Meanwhile, among the 365 healthy controls, 26 presented the cysteine‐sparing *NOTCH3* mutations (Table S2). Therefore, the cysteine‐sparing *NOTCH3* variants were not enriched in the SVaD patients without cysteine‐altering *NOTCH3* variant when compared to controls.

### 3.6 Association analysis between *NOTCH3* variants and AD

In the single‐variant‐based analysis, 53 common variants met the criteria for further association analysis: genotyping rate >80% and *p* value of Hardy–Weinberg test >0.05. None of the common variants reached statistical significance between AD patients and controls after the adjustment of age, gender, and *APOE* ε4 status (*p *> 0.05; Table S3). In the gene‐based rare missense variants analysis, 53 rare missense variants were collapsed together (Table S4). Thirty‐two variants were present in AD patients only, 11 were only detected in the controls, and the remaining 10 were identified in both AD patients and controls. No significant enrichment of *NOTCH3* missense variants was detected in AD cases when compared to controls (10.2% vs 7.1%, *p* = 0.21).

## 4 DISCUSSION

Among the 96 SVaD patients in our cohort, 16 (16.7%) carried a *NOTCH3* pathogenic mutation, suggesting that *NOTCH3* pathogenic mutations were common in SVaD. A previous study in South Korea investigating the *NOTCH3* variants in 117 patients with subcortical vascular cognitive impairment showed that three pathogenic mutations were found in eight patients. The high prevalence of *NOTCH3* pathogenic mutations in both studies implied that CADASIL was an important etiology for vascular cognitive impairment. It is necessary to screen *NOTCH3* in patients diagnosed with vascular cognitive impairment, especially for those with a positive history. No pathogenic mutation in *NOTCH3* was detected in AD patients, suggesting that *NOTCH3* pathogenic mutation was rare in patients clinically diagnosed with AD. Although a *NOTCH3* pathogenic mutation was identified in a Turkish AD family, this might have been caused by the clinical heterogeneity of CADASIL or the coexistence of CADASIL and AD, because the patient was diagnosed with AD clinically rather than pathologically.^12^

Both of the two novel likely pathogenic mutations, p.C201F and p.C1061F, replaced a cysteine residue with phenylalanine. Three mutations affecting the codon 201 (p.C201Y, p.C201R, and p.C201S), and a mutation changing the amino acid at position 1061(p.C1061Y), have been reported as pathogenic mutations of CADASIL.^28^, ^29^, ^30^, ^31^ The two novel mutations led to the loss of a cysteine residue in the EGFr 5 and EGFr 27, respectively. In the three patients with a mutation in the EGFr 4 or 5, cognitive or psychiatric symptoms occurred around age 50 and deteriorated quickly. This was consistent with the research conducted by Rutten et al. that *NOTCH3* EGFr 1–6 pathogenic mutations are associated with a more severe phenotype than EGFr 7–34 pathogenic mutations.^32^

The most common pathogenic mutation in our cohort was p.R544C, followed by p.R607C. Nevertheless, a recent study investigating *NOTCH3* variants in 261 Chinese patients with clinically suspected CADASIL showed that p.R607C and p.R544C were the first and second most common variants, respectively.^30^ The discrepancy in the mutational spectrum may be explained by the phenotypes difference of different mutations. Since patients with p.R607C tend to present a more classical CADASIL phenotype than those with p.R544C, and in our study we have only included patients with dementia caused by SVaD and AD. The seven probands carrying the p.R544C mutation started with cognitive decline after the age of 60, except for patient 6, and none of them experienced a migraine. These results were consistent with previous findings indicating that patients with the p.R544C mutation predominantly present a late‐onset disease with a mild and atypical phenotype, such as the rare occurrence of migraine and a low frequency of WMH in the temporal pole^33^; however, the specific mechanism behind these observations has not been identified. Some studies speculate that the different effects of the mutations on NOTCH3 signaling may be involved in modifying the CADASIL phenotype.^34^ The majority of the mutations in our cohort were in exon 11 of the *NOTCH3* gene, in accordance with previous studies indicating that exon 11 is a hot region in the Chinese population,^30^ while exon 4 is currently the most common mutated exon in the literature.^9^, ^35^

Our findings showed that SVaD patients with *NOTCH3* mutations had an earlier age of onset and a higher frequency of cognitive impairment in their family history than those without *NOTCH3* mutations. However, a previous study did not find differences in the clinical features of patients with and without *NOTCH3* variants that presented subcortical vascular impairment. We speculated that the differences observed were mainly caused by cysteine‐altering mutations, because typical CADASIL mutations tend to present similar characteristics and the proportion of typical CADASIL mutations found in our study was higher than that reported by Yoon et al.^8^

To our surprise, six cysteine‐sparing mutations previously reported in suspected CADASIL patients were detected in 10 controls, which raised the question whether these were causative mutations of CADASIL. The mutation p.G1347R was first reported in a CADASIL‐like case with chronic renal failure and suspicious GOM in the renal arterioles.^22^ However, most of the mutation carriers in our cohort presented typical AD phenotype, and no GOM deposition was observed in the AD patient with the most significant vascular lesions on MRI. We then reviewed the original literature and believed that the suspected GOM alleged by the author was an immune complex and the non‐cysteine substitution could not explain the WMH on MRI. The mutation p.P167S was first reported in a Japanese CADASIL patient in 2006,^26^ but a typical pathogenic mutation (p.R182C) was detected in the same patient in further genetic testing several years later, and p.R182C was co‐segregated in the family rather than p.P167S.^36^ This implicated that p.P167S was not a causative mutation. More and more recent investigations have found that *NOTCH3* cysteine‐sparing mutations were not rare and questioned the pathogenicity of this type of mutations. Rutten et al. studied 11 *NOTCH3* missense mutations that did not involve a cysteine residue and concluded that there was no compelling evidence that these mutations were associated with CADASIL, except for p.R75P.^9^ Subsequently, Muiño et al. conducted a systematic review of cysteine‐sparing *NOTCH3* missense mutations in clinically suspected CADASIL patients in 2017 and believed that only four (p.R61W, p.R75P, p.D80G, and p.R213K) were possible pathogenic mutations, while the remaining 21 cysteine‐sparing mutations, including p.V237M and p.A1020P, lacked convincing evidence of pathogenicity because of atypical CADASIL phenotype, relatively high frequency in a public database, no GOM deposition in the skin biopsy, or other pathogenic mutations that cannot be ruled out due to incomplete exons sequencing.^11^ When symmetrical WMH on brain MRI is present in a patient with recurrent migraine or stroke attacks or inexplicable cognitive impairment, CADASIL ought to be considered, but a *NOTCH3* cysteine‐sparing mutation should not be interpreted as a pathogenic variant unless sufficient genetic and clinical evidence has been obtained.

AD and VaD are the two most common types of dementia,^37^ and CADASIL is a common cause of VaD. Although the pathogenesis was different, AD and CADASIL had many similarities in pathology. Both AD and CADASIL are characterized by the aggregation of abnormal proteins, and in both cases, the deposited proteins were cleaved by gamma‐secretase.^38^ Although recent studies showed that variations in the *NOTCH3* gene were correlated with AD in the western population, we found that neither common variants nor rare missense variants in *NOTCH3* were associated with AD in the Chinese population. The possible reasons are as follows: First, the relatively small sample size in our research (only 365 controls) may lead to a negative result in association analysis, particularly for rare variants; second, the inclusion and exclusion criteria varied among different studies; third, racial differences may account for the inconsistencies in the association analysis between *NOTCH3* gene variants and AD; and finally, our targeted capture sequencing mainly focused on variants in the coding regions and adjacent intron regions of *NOTCH3*, while most known AD risk loci are in non‐coding regions. Therefore, further investigation is necessary to study the relationship between *NOTCH3* and AD in a larger cohort, including variants in both the coding and non‐coding regions.

In conclusion, this is the first systematic study of *NOTCH3* variants in a large Chinese cohort of AD and SVaD, and two novel likely pathogenic mutations, p.C201F and p.C1061F, were identified. Pathogenic mutations in *NOTCH3* are relatively common in SVaD patients. In addition, we believe that six cysteine‐sparing mutations previously reported in CADASIL patients were rare polymorphisms rather than CADASIL causative mutations, suggesting that the pathogenicity of cysteine‐sparing mutations in *NOTCH3* should be interpreted prudently. Our association study showed that *NOTCH3* was not related to AD in the Chinese population whether from the perspective of common variants or the perspective of gene‐based rare mutations; more studies are necessary to further elucidate this finding.

## CONFLICT OF INTEREST

The authors report no actual or potential conflicts of interest.

## Supporting information

Supplementary Material

## Data Availability

The data that support the findings of this study are available from the corresponding author upon reasonable request.
