# Supplementary material for: The role of NOTCH3 variants in Alzheimer's disease and subcortical vascular dementia in the Chinese population
Source: CNS Neurosci Ther. 2021 May 4;27(8):930–40. doi: 10.1111/cns.13647 (PMC8265940; doi:10.1111/cns.13647)

**Supplementary Table S1. 345 variants in NOTCH3 identified in 667 AD cases, 95 SVaD cases, and 365 healthy elderly controls.**

| **NO.** | **Rare/**  **Common** | **Start** | **End** | **Ref** | **Alt** | **Function region** | **Nucleotide change and amino acid change** |
| --- | --- | --- | --- | --- | --- | --- | --- |
| 1 | Rare | chr19:15270294 | chr19:15270294 | G | T | UTR3 |  |
| 2 | Rare | chr19:15270306 | chr19:15270306 | C | T | UTR3 |  |
| 3 | Rare | chr19:15270342 | chr19:15270342 | T | A | UTR3 |  |
| 4 | Rare | chr19:15270417 | chr19:15270417 | C | A | UTR3 |  |
| 5 | Rare | chr19:15270422 | chr19:15270422 | C | T | UTR3 |  |
| 6 | Common | chr19:15270536 | chr19:15270536 | A | G | UTR3 |  |
| 7 | Common | chr19:15270583 | chr19:15270583 | G | A | UTR3 |  |
| 8 | Common | chr19:15270636 | chr19:15270636 | C | T | UTR3 |  |
| 9 | Rare | chr19:15270642 | chr19:15270642 | G | C | UTR3 |  |
| 10 | Common | chr19:15270665 | chr19:15270665 | C | T | UTR3 |  |
| 11 | Rare | chr19:15270800 | chr19:15270800 | T | C | UTR3 |  |
| 12 | Common | chr19:15270805 | chr19:15270805 | C | A | UTR3 |  |
| 13 | Rare | chr19:15270832 | chr19:15270832 | G | A | UTR3 |  |
| 14 | Rare | chr19:15270844 | chr19:15270844 | G | A | UTR3 |  |
| 15 | Rare | chr19:15270864 | chr19:15270864 | C | T | UTR3 |  |
| 16 | Rare | chr19:15270895 | chr19:15270895 | G | A | UTR3 |  |
| 17 | Rare | chr19:15271028 | chr19:15271028 | C | T | UTR3 |  |
| 18 | Rare | chr19:15271071 | chr19:15271071 | T | A | UTR3 |  |
| 19 | Rare | chr19:15271089 | chr19:15271089 | T | A | UTR3 |  |
| 20 | Rare | chr19:15271090 | chr19:15271090 | T | A | UTR3 |  |
| 21 | Rare | chr19:15271116 | chr19:15271116 | G | T | UTR3 |  |
| 22 | Common | chr19:15271117 | chr19:15271117 | - | A | UTR3 |  |
| 23 | Common | chr19:15271135 | chr19:15271135 | T | G | UTR3 |  |
| 24 | Rare | chr19:15271354 | chr19:15271354 | G | A | UTR3 |  |
| 25 | Rare | chr19:15271437 | chr19:15271437 | C | G | UTR3 |  |
| 26 | Rare | chr19:15271566 | chr19:15271566 | T | G | exonic | *NOTCH3*:NM_000435:exon33:c.6873A>C:p.P2291P |
| 27 | Rare | chr19:15271654 | chr19:15271654 | G | A | exonic | *NOTCH3*:NM_000435:exon33:c.6785C>T:p.S2262F |
| 28 | Rare | chr19:15271770 | chr19:15271770 | C | A | exonic | *NOTCH3*:NM_000435:exon33:c.6669G>T:p.A2223A |
| 29 | Common | chr19:15271771 | chr19:15271771 | G | A | exonic | *NOTCH3*:NM_000435:exon33:c.6668C>T:p.A2223V |
| 30 | Rare | chr19:15271785 | chr19:15271785 | G | A | exonic | *NOTCH3*:NM_000435:exon33:c.6654C>T:p.G2218G |
| 31 | Rare | chr19:15271796 | chr19:15271796 | G | C | exonic | *NOTCH3*:NM_000435:exon33:c.6643C>G:p.P2215A |
| 32 | Rare | chr19:15271812 | chr19:15271812 | C | T | exonic | *NOTCH3*:NM_000435:exon33:c.6627G>A:p.P2209P |
| 33 | Rare | chr19:15271813 | chr19:15271813 | G | A | exonic | *NOTCH3*:NM_000435:exon33:c.6626C>T:p.P2209L |
| 34 | Rare | chr19:15271818 | chr19:15271818 | C | T | exonic | *NOTCH3*:NM_000435:exon33:c.6621G>A:p.R2207R |
| 35 | Rare | chr19:15271819 | chr19:15271819 | C | T | exonic | *NOTCH3*:NM_000435:exon33:c.6620G>A:p.R2207Q |
| 36 | Rare | chr19:15271820 | chr19:15271820 | G | A | exonic | *NOTCH3*:NM_000435:exon33:c.6619C>T:p.R2207W |
| 37 | Rare | chr19:15271867 | chr19:15271867 | G | A | exonic | *NOTCH3*:NM_000435:exon33:c.6572C>T:p.P2191L |
| 38 | Rare | chr19:15271959 | chr19:15271959 | C | T | exonic | *NOTCH3*:NM_000435.2:exon33:c.6480G>A:p.L2160L |
| 39 | Common | chr19:15272001 | chr19:15272001 | C | T | exonic | *NOTCH3*:NM_000435:exon33:c.6438G>A:p.A2146A |
| 40 | Rare | chr19:15272005 | chr19:15272005 | C | T | exonic | *NOTCH3*:NM_000435exon33:c.6434G>A:p.R2145Q |
| 41 | Rare | chr19:15272127 | chr19:15272127 | C | T | exonic | *NOTCH3*:NM_000435:exon33:c.6312G>A:p.S2104S |
| 42 | Rare | chr19:15272145 | chr19:15272145 | C | T | exonic | *NOTCH3*:NM_000435:exon33:c.6294G>A:p.T2098T |
| 43 | Rare | chr19:15272151 | chr19:15272151 | C | T | exonic | *NOTCH3*:NM_000435:exon33:c.6288G>A:p.S2096S |
| 44 | Rare | chr19:15272239 | chr19:15272239 | G | A | exonic | *NOTCH3*:NM_000435:exon33:c.6200C>T:p.P2067L |
| 45 | Rare | chr19:15272339 | chr19:15272339 | G | A | exonic | *NOTCH3*:NM_000435:exon33:c.6100C>T:p.P2034S |
| 46 | Rare | chr19:15272436 | chr19:15272436 | G | A | exonic | *NOTCH3*:NM_000435:exon33:c.6003C>T:p.T2001T |
| 47 | Rare | chr19:15272502 | chr19:15272502 | G | A | exonic | *NOTCH3*:NM_000435:exon33:c.5937C>T:p.A1979A |
| 48 | Rare | chr19:15272612 | chr19:15272612 | T | C | intronic |  |
| 49 | Rare | chr19:15272614 | chr19:15272614 | A | T | intronic |  |
| 50 | Rare | chr19:15272623 | chr19:15272623 | A | T | intronic |  |
| 51 | Rare | chr19:15272667 | chr19:15272667 | G | A | intronic |  |
| 52 | Rare | chr19:15273047 | chr19:15273047 | C | T | intronic |  |
| 53 | Rare | chr19:15273097 | chr19:15273097 | G | A | intronic |  |
| 54 | Rare | chr19:15273194 | chr19:15273194 | G | C | intronic |  |
| 55 | Common | chr19:15273221 | chr19:15273221 | T | C | intronic |  |
| 56 | Rare | chr19:15273227 | chr19:15273227 | T | A | intronic |  |
| 57 | Common | chr19:15273231 | chr19:15273231 | A | G | intronic |  |
| 58 | Common | chr19:15273248 | chr19:15273248 | A | C | intronic |  |
| 59 | Rare | chr19:15273288 | chr19:15273288 | C | T | exonic | *NOTCH3*:NM_000435:exon32:c.5901G>A:p.M1967I |
| 60 | Rare | chr19:15273335 | chr19:15273335 | C | T | exonic | *NOTCH3*:NM_000435:exon32:c.5854G>A:p.V1952M |
| 61 | Rare | chr19:15273357 | chr19:15273357 | G | A | exonic | *NOTCH3*:NM_000435:exon32:c.5832C>T:p.H1944H |
| 62 | Common | chr19:15273381 | chr19:15273381 | A | G | intronic |  |
| 63 | Rare | chr19:15273382 | chr19:15273382 | T | C | intronic |  |
| 64 | Rare | chr19:15273407 | chr19:15273407 | G | T | intronic |  |
| 65 | Rare | chr19:15273432 | chr19:15273432 | A | C | intronic |  |
| 66 | Rare | chr19:15273451 | chr19:15273451 | A | G | intronic |  |
| 67 | Rare | chr19:15273539 | chr19:15273539 | T | C | intronic |  |
| 68 | Rare | chr19:15273556 | chr19:15273556 | G | A | intronic |  |
| 69 | Rare | chr19:15273576 | chr19:15273576 | G | C | intronic |  |
| 70 | common | chr19:15273583 | chr19:15273584 | TT | - | intronic |  |
| 71 | Common | chr19:15273584 | chr19:15273584 | T | G | intronic |  |
| 72 | Rare | chr19:15273584 | chr19:15273584 | T | A | intronic |  |
| 73 | Common | chr19:15273588 | chr19:15273588 | G | A | intronic |  |
| 74 | common | chr19:15273624 | chr19:15273624 | T | C | intronic |  |
| 75 | Common | chr19:15276007 | chr19:15276007 | C | A | intronic |  |
| 76 | Common | chr19:15276055 | chr19:15276055 | C | G | intronic |  |
| 77 | Rare | chr19:15276139 | chr19:15276139 | A | G | intronic |  |
| 78 | Common | chr19:15276143 | chr19:15276143 | C | T | intronic |  |
| 79 | Rare | chr19:15276193 | chr19:15276193 | G | A | exonic | *NOTCH3*:NM_000435:exon31:c.5801C>T:p.A1934V |
| 80 | Rare | chr19:15276230 | chr19:15276230 | C | A | exonic | *NOTCH3*:NM_000435exon31:c.5764G>T:p.V1922L |
| 81 | Rare | chr19:15276255 | chr19:15276255 | C | T | exonic | *NOTCH3*:NM_000435:exon31:c.5739G>A:p.A1913A |
| 82 | Rare | chr19:15276424 | chr19:15276424 | A | C | intronic |  |
| 83 | Rare | chr19:15276430 | chr19:15276430 | T | C | intronic |  |
| 84 | Rare | chr19:15276451 | chr19:15276451 | A | G | intronic |  |
| 85 | Rare | chr19:15276575 | chr19:15276575 | T | A | intronic |  |
| 86 | Rare | chr19:15276717 | chr19:15276717 | C | T | exonic | *NOTCH3*:NM_000435:exon30:c.5548G>A:p.A1850T |
| 87 | Rare | chr19:15276755 | chr19:15276755 | C | T | exonic | *NOTCH3*:NM_000435:exon30:c.5510G>A:p.R1837H |
| 88 | Rare | chr19:15276756 | chr19:15276756 | G | A | exonic | *NOTCH3*:NM_000435:exon30:c.5509C>T:p.R1837C |
| 89 | Rare | chr19:15276805 | chr19:15276805 | G | T | exonic | *NOTCH3*:NM_000435:exon30:c.5460C>A:p.I1820I |
| 90 | Rare | chr19:15276863 | chr19:15276863 | C | T | exonic | *NOTCH3*:NM_000435:exon30:c.5402G>A:p.G1801E |
| 91 | Common | chr19:15276919 | chr19:15276919 | G | A | intronic |  |
| 92 | Common | chr19:15276923 | chr19:15276923 | C | T | intronic |  |
| 93 | Rare | chr19:15276999 | chr19:15276999 | G | A | intronic |  |
| 94 | Common | chr19:15277006 | chr19:15277006 | T | C | intronic |  |
| 95 | Common | chr19:15277009 | chr19:15277009 | C | T | intronic |  |
| 96 | Common | chr19:15277010 | chr19:15277010 | A | G | intronic |  |
| 97 | Rare | chr19:15277021 | chr19:15277021 | C | T | intronic |  |
| 98 | Rare | chr19:15277022 | chr19:15277022 | C | T | intronic |  |
| 99 | Common | chr19:15277027 | chr19:15277027 | G | A | intronic |  |
| 100 | Rare | chr19:15277039 | chr19:15277039 | A | G | intronic |  |
| 101 | Rare | chr19:15277065 | chr19:15277065 | C | T | intronic |  |
| 102 | common | chr19:15277199 | chr19:15277199 | C | T | intronic |  |
| 103 | Common | chr19:15277938 | chr19:15277938 | C | T | intronic |  |
| 104 | Rare | chr19:15277940 | chr19:15277940 | C | T | intronic |  |
| 105 | Rare | chr19:15277965 | chr19:15277965 | C | A | intronic |  |
| 106 | Common | chr19:15278057 | chr19:15278057 | A | G | intronic |  |
| 107 | Rare | chr19:15278069 | chr19:15278069 | G | A | exonic | *NOTCH3*:NM_000435:exon29:c.5353C>T:p.R1785C |
| 108 | Rare | chr19:15278262 | chr19:15278262 | T | C | intronic |  |
| 109 | Common | chr19:15278335 | chr19:15278335 | G | T | intronic |  |
| 110 | Rare | chr19:15278337 | chr19:15278337 | G | A | intronic |  |
| 111 | common | chr19:15278373 | chr19:15278373 | - | TTTG | intronic |  |
| 112 | Rare | chr19:15278404 | chr19:15278404 | C | A | intronic |  |
| 113 | Rare | chr19:15278406 | chr19:15278406 | C | T | intronic |  |
| 114 | Rare | chr19:15278409 | chr19:15278409 | G | A | intronic |  |
| 115 | Rare | chr19:15278519 | chr19:15278519 | G | T | intronic |  |
| 116 | Rare | chr19:15280699 | chr19:15280699 | G | T | intronic |  |
| 117 | Rare | chr19:15280706 | chr19:15280706 | C | A | intronic |  |
| 118 | Common | chr19:15280765 | chr19:15280765 | A | G | intronic |  |
| 119 | Rare | chr19:15280786 | chr19:15280786 | G | A | intronic |  |
| 120 | Rare | chr19:15280826 | chr19:15280826 | C | G | intronic |  |
| 121 | Rare | chr19:15280886 | chr19:15280886 | G | T | intronic |  |
| 122 | Rare | chr19:15280910 | chr19:15280910 | G | A | exonic | *NOTCH3*:NM_000435:exon28:c.5186C>T:p.A1729V |
| 123 | Rare | chr19:15281108 | chr19:15281108 | G | A | intronic |  |
| 124 | Rare | chr19:15281116 | chr19:15281116 | G | A | intronic |  |
| 125 | Rare | chr19:15281174 | chr19:15281174 | C | T | exonic | *NOTCH3*:NM_000435:exon27:c.5082G>A:p.R1694R |
| 126 | Rare | chr19:15281175 | chr19:15281175 | C | T | exonic | *NOTCH3*:NM_000435:exon27:c.5081G>A:p.R1694Q |
| 127 | Rare | chr19:15281185 | chr19:15281185 | T | C | exonic | *NOTCH3*:NM_000435:exon27:c.5071A>G:p.K1691E |
| 128 | Rare | chr19:15281281 | chr19:15281281 | C | T | exonic | *NOTCH3*:NM_000435:exon27:c.4975G>A:p.V1659I |
| 129 | Rare | chr19:15281357 | chr19:15281357 | C | G | exonic | *NOTCH3*:NM_000435:exon27:c.4899G>C:p.P1633P |
| 130 | Common | chr19:15281386 | chr19:15281386 | C | A | intronic |  |
| 131 | Rare | chr19:15281405 | chr19:15281405 | G | T | intronic |  |
| 132 | rare | chr19:15281435 | chr19:15281435 | G | A | intronic |  |
| 133 | Common | chr19:15281459 | chr19:15281459 | C | G | intronic |  |
| 134 | Rare | chr19:15281534 | chr19:15281534 | C | G | exonic | *NOTCH3*:NM_000435:exon26:c.4839G>C:p.A1613A |
| 135 | Rare | chr19:15281580 | chr19:15281580 | T | A | exonic | *NOTCH3*:NM_000435:exon26:c.4793A>T:p.D1598V |
| 136 | Rare | chr19:15281582 | chr19:15281582 | A | T | exonic | *NOTCH3*:NM_000435:exon26:c.4791T>A:p.N1597K |
| 137 | Rare | chr19:15281591 | chr19:15281591 | C | G | exonic | *NOTCH3*:NM_000435:exon26:c.4782G>C:p.S1594S |
| 138 | rare | chr19:15281686 | chr19:15281686 | - | C | intronic |  |
| 139 | Rare | chr19:15281707 | chr19:15281707 | C | A | intronic |  |
| 140 | Rare | chr19:15281844 | chr19:15281844 | A | G | intronic |  |
| 141 | Rare | chr19:15284668 | chr19:15284668 | G | A | intronic |  |
| 142 | Rare | chr19:15284759 | chr19:15284759 | G | T | intronic |  |
| 143 | Rare | chr19:15284790 | chr19:15284790 | C | T | intronic |  |
| 144 | Rare | chr19:15284793 | chr19:15284793 | T | C | intronic |  |
| 145 | Rare | chr19:15284818 | chr19:15284818 | T | C | intronic |  |
| 146 | Rare | chr19:15284909 | chr19:15284909 | G | A | exonic | *NOTCH3*:NM_000435:exon25:c.4706C>T:p.A1569V |
| 147 | Rare | chr19:15285000 | chr19:15285000 | G | A | exonic | *NOTCH3*:NM_000435:exon25:c.4615C>T:p.L1539L |
| 148 | Common | chr19:15285052 | chr19:15285052 | T | C | exonic | *NOTCH3*:NM_000435:exon25:c.4563A>G:p.P1521P |
| 149 | Common | chr19:15285063 | chr19:15285063 | G | T | exonic | *NOTCH3*:NM_000435:exon25:c.4552C>A:p.L1518M |
| 150 | Rare | chr19:15285251 | chr19:15285251 | G | T | intronic |  |
| 151 | Rare | chr19:15285253 | chr19:15285253 | T | C | intronic |  |
| 152 | Common | chr19:15285272 | chr19:15285272 | T | C | intronic |  |
| 153 | rare | chr19:15285383 | chr19:15285383 | T | - | intronic |  |
| 154 | rare | chr19:15285386 | chr19:15285386 | T | - | intronic |  |
| 155 | Rare | chr19:15285392 | chr19:15285392 | T | C | intronic |  |
| 156 | Rare | chr19:15285421 | chr19:15285421 | C | G | intronic |  |
| 157 | Common | chr19:15285422 | chr19:15285422 | G | A | intronic |  |
| 158 | common | chr19:15285542 | chr19:15285542 | G | T | intronic |  |
| 159 | rare | chr19:15288063 | chr19:15288063 | A | - | intronic |  |
| 160 | Rare | chr19:15288231 | chr19:15288231 | A | G | intronic |  |
| 161 | Rare | chr19:15288260 | chr19:15288260 | A | G | intronic |  |
| 162 | Rare | chr19:15288391 | chr19:15288391 | C | T | exonic | *NOTCH3*:NM_000435:exon24:c.4348G>A:p.A1450T |
| 163 | Rare | chr19:15288568 | chr19:15288568 | C | G | exonic | *NOTCH3*:NM_000435:exon24:c.4171G>C:p.A1391P |
| 164 | Rare | chr19:15288653 | chr19:15288653 | A | G | exonic | *NOTCH3*:NM_000435:exon24:c.4086T>C:p.A1362A |
| 165 | Rare | chr19:15288678 | chr19:15288678 | G | A | exonic | *NOTCH3*:NM_000435:exon24:c.4061C>T:p.P1354L _†_ |
| 166 | Rare | chr19:15288700 | chr19:15288700 | C | G | exonic | *NOTCH3*:NM_000435:exon24:c.4039G>C:p.G1347R _‡_ |
| 167 | Rare | chr19:15288793 | chr19:15288793 | G | A | exonic | *NOTCH3*:NM_000435:exon24:c.3946C>T:p.P1316S |
| 168 | Rare | chr19:15288922 | chr19:15288922 | G | C | intronic |  |
| 169 | Common | chr19:15288926 | chr19:15288926 | G | A | intronic |  |
| 170 | Rare | chr19:15289000 | chr19:15289000 | G | A | intronic |  |
| 171 | Rare | chr19:15289007 | chr19:15289007 | G | A | intronic |  |
| 172 | Rare | chr19:15289448 | chr19:15289448 | G | A | intronic |  |
| 173 | Rare | chr19:15289550 | chr19:15289550 | A | G | intronic |  |
| 174 | Rare | chr19:15289557 | chr19:15289557 | C | T | intronic |  |
| 175 | Rare | chr19:15289612 | chr19:15289612 | A | G | intronic |  |
| 176 | Common | chr19:15289613 | chr19:15289613 | A | T | intronic |  |
| 177 | Common | chr19:15289763 | chr19:15289763 | G | A | intronic |  |
| 178 | Rare | chr19:15289936 | chr19:15289936 | G | A | exonic | *NOTCH3*:NM_000435:exon22:c.3618C>T:p.I1206I |
| 179 | Rare | chr19:15289994 | chr19:15289994 | C | T | exonic | *NOTCH3*:NM_000435:exon22:c.3560G>A:p.G1187D |
| 180 | Rare | chr19:15290030 | chr19:15290030 | C | T | exonic | *NOTCH3*:NM_000435:exon22:c.3524G>A:p.R1175Q |
| 181 | Common | chr19:15290031 | chr19:15290031 | G | A | exonic | *NOTCH3*:NM_000435:exon22:c.3523C>T:p.R1175W |
| 182 | Common | chr19:15290125 | chr19:15290125 | G | A | ncRNA_exonic |  |
| 183 | Rare | chr19:15290168 | chr19:15290168 | G | A | intronic |  |
| 184 | Rare | chr19:15290180 | chr19:15290180 | G | A | exonic | *NOTCH3*:NM_000435:exon21:c.3455C>T:p.T1152M |
| 185 | Rare | chr19:15290278 | chr19:15290278 | A | G | exonic | *NOTCH3*:NM_000435:exon21:c.3357T>C:p.C1119C |
| 186 | Rare | chr19:15290339 | chr19:15290339 | C | G | intronic |  |
| 187 | Rare | chr19:15290345 | chr19:15290345 | G | A | intronic |  |
| 188 | Rare | chr19:15290406 | chr19:15290406 | C | T | intronic |  |
| 189 | Common | chr19:15290412 | chr19:15290412 | G | A | intronic |  |
| 190 | Rare | chr19:15290459 | chr19:15290459 | G | A | intronic |  |
| 191 | Rare | chr19:15290465 | chr19:15290465 | G | A | intronic |  |
| 192 | Rare | chr19:15290491 | chr19:15290491 | G | A | intronic |  |
| 193 | rare | chr19:15290769 | chr19:15290769 | A | G | intronic |  |
| 194 | Rare | chr19:15290775 | chr19:15290775 | T | C | intronic |  |
| 195 | Rare | chr19:15290871 | chr19:15290871 | G | T | intronic |  |
| 196 | Rare | chr19:15290872 | chr19:15290872 | C | T | intronic |  |
| 197 | Rare | chr19:15290873 | chr19:15290873 | G | A | intronic |  |
| 198 | Rare | chr19:15290911 | chr19:15290911 | C | T | exonic | *NOTCH3*:NM_000435:exon20:c.3299G>A:p.R1100H |
| 199 | Rare | chr19:15290950 | chr19:15290950 | G | A | exonic | *NOTCH3*:NM_000435:exon20:c.3260C>T:p.P1087L |
| 200 | Rare | chr19:15291028 | chr19:15291028 | C | A | exonic | *NOTCH3*:NM_000435:exon20:c.3182G>T:p.C1061F |
| 201 | Common | chr19:15291096 | chr19:15291096 | G | A | intronic |  |
| 202 | Common | chr19:15291136 | chr19:15291136 | G | A | intronic |  |
| 203 | Rare | chr19:15291226 | chr19:15291226 | A | T | intronic |  |
| 204 | Rare | chr19:15291252 | chr19:15291252 | C | T | intronic |  |
| 205 | Rare | chr19:15291269 | chr19:15291269 | C | T | intronic |  |
| 206 | Rare | chr19:15291382 | chr19:15291382 | A | G | intronic |  |
| 207 | Rare | chr19:15291416 | chr19:15291416 | G | A | intronic |  |
| 208 | Rare | chr19:15291437 | chr19:15291437 | C | A | intronic |  |
| 209 | Rare | chr19:15291438 | chr19:15291438 | G | A | intronic |  |
| 210 | rare | chr19:15291460 | chr19:15291460 | C | T | intronic |  |
| 211 | Rare | chr19:15291538 | chr19:15291538 | G | A | exonic | *NOTCH3*:NM_000435:exon19:c.3096C>T:p.L1032L |
| 212 | Common | chr19:15291576 | chr19:15291576 | C | G | exonic | *NOTCH3*:NM_000435:exon19:c.3058G>C:p.A1020P |
| 213 | Rare | chr19:15291618 | chr19:15291618 | G | A | exonic | *NOTCH3*:NM_000435:exon19:c.3016C>T:p.R1006C |
| 214 | Rare | chr19:15291676 | chr19:15291676 | G | T | intronic |  |
| 215 | Common | chr19:15291699 | chr19:15291699 | T | C | intronic |  |
| 216 | Rare | chr19:15291708 | chr19:15291708 | T | A | intronic |  |
| 217 | Rare | chr19:15291783 | chr19:15291783 | G | A | exonic | *NOTCH3*:NM_000435:exon18:c.2983C>T:p.P995S |
| 218 | Rare | chr19:15291860 | chr19:15291860 | C | T | exonic | *NOTCH3*:NM_000435:exon18:c.2906G>A:p.R969Q |
| 219 | Rare | chr19:15291919 | chr19:15291919 | C | T | exonic | *NOTCH3*:NM_000435:exon18:c.2847G>A:p.L949L |
| 220 | Common | chr19:15292088 | chr19:15292088 | G | C | intronic |  |
| 221 | Rare | chr19:15292091 | chr19:15292091 | A | C | intronic |  |
| 222 | Common | chr19:15292271 | chr19:15292271 | C | T | intronic |  |
| 223 | Common | chr19:15292365 | chr19:15292365 | T | A | intronic |  |
| 224 | Common | chr19:15292366 | chr19:15292366 | C | T | intronic |  |
| 225 | Common | chr19:15292437 | chr19:15292437 | T | C | exonic | *NOTCH3*:NM_000435:exon17:c.2742A>G:p.P914P |
| 226 | Common | chr19:15292599 | chr19:15292599 | G | A | exonic | *NOTCH3*:NM_000435:exon17:c.2580C>T:p.N860N |
| 227 | Rare | chr19:15292616 | chr19:15292616 | C | T | intronic |  |
| 228 | Rare | chr19:15294889 | chr19:15294889 | A | G | intronic |  |
| 229 | Rare | chr19:15294924 | chr19:15294924 | C | T | intronic |  |
| 230 | Rare | chr19:15294927 | chr19:15294927 | G | A | intronic |  |
| 231 | Rare | chr19:15294944 | chr19:15294944 | A | G | intronic |  |
| 232 | Common | chr19:15294991 | chr19:15294991 | C | T | intronic |  |
| 233 | Rare | chr19:15295041 | chr19:15295041 | A | C | intronic |  |
| 234 | Common | chr19:15295134 | chr19:15295134 | G | A | exonic | *NOTCH3*:NM_000435:exon16:c.2538C>T:p.C846C |
| 235 | Rare | chr19:15295349 | chr19:15295349 | C | T | intronic |  |
| 236 | Rare | chr19:15295406 | chr19:15295406 | A | T | intronic |  |
| 237 | Rare | chr19:15295469 | chr19:15295469 | C | T | intronic |  |
| 238 | Rare | chr19:15295564 | chr19:15295564 | C | T | intronic |  |
| 239 | Rare | chr19:15295613 | chr19:15295613 | G | A | intronic |  |
| 240 | Rare | chr19:15295900 | chr19:15295900 | T | A | intronic |  |
| 241 | Rare | chr19:15295955 | chr19:15295955 | A | C | intronic |  |
| 242 | Rare | chr19:15296033 | chr19:15296033 | T | C | intronic |  |
| 243 | Rare | chr19:15296081 | chr19:15296081 | C | G | exonic | *NOTCH3*:NM_000435:exon14:c.2283G>C:p.P761P |
| 244 | Rare | chr19:15296129 | chr19:15296129 | G | A | exonic | *NOTCH3*:NM_000435:exon14:c.2235C>T:p.A745A |
| 245 | Rare | chr19:15296130 | chr19:15296130 | G | A | exonic | *NOTCH3*:NM_000435:exon14:c.2234C>T:p.A745V |
| 246 | Rare | chr19:15296186 | chr19:15296186 | G | A | exonic | *NOTCH3*:NM_000435:exon14:c.2178C>T:p.G726G |
| 247 | Rare | chr19:15296441 | chr19:15296441 | G | A | exonic | *NOTCH3*:NM_000435:exon13:c.2001C>T:p.G667G |
| 248 | Rare | chr19:15296513 | chr19:15296513 | C | T | intronic |  |
| 249 | Rare | chr19:15296522 | chr19:15296522 | A | C | intronic |  |
| 250 | Rare | chr19:15296584 | chr19:15296584 | C | T | intronic |  |
| 251 | Rare | chr19:15296639 | chr19:15296639 | G | A | intronic |  |
| 252 | Rare | chr19:15296676 | chr19:15296676 | C | T | intronic |  |
| 253 | Rare | chr19:15296680 | chr19:15296680 | T | G | intronic |  |
| 254 | common | chr19:15296717 | chr19:15296717 | C | T | intronic |  |
| 255 | Rare | chr19:15297481 | chr19:15297481 | G | A | intronic |  |
| 256 | Rare | chr19:15297508 | chr19:15297508 | G | A | intronic |  |
| 257 | Rare | chr19:15297512 | chr19:15297512 | A | G | intronic |  |
| 258 | Common | chr19:15297576 | chr19:15297576 | C | T | intronic |  |
| 259 | Rare | chr19:15297671 | chr19:15297671 | T | C | intronic |  |
| 260 | Rare | chr19:15297937 | chr19:15297937 | G | A | exonic | *NOTCH3*:NM_000435:exon11:c.1819C>T:p.R607C |
| 261 | Rare | chr19:15297978 | chr19:15297978 | T | C | exonic | *NOTCH3*:NM_000435:exon11:c.1778A>G:p.H593R |
| 262 | Rare | chr19:15298031 | chr19:15298031 | C | T | exonic | *NOTCH3*:NM_000435:exon11:c.1725G>A:p.T575T |
| 263 | Rare | chr19:15298064 | chr19:15298064 | G | A | exonic | *NOTCH3*:NM_000435:exon11:c.1692C>T:p.A564A |
| 264 | Rare | chr19:15298066 | chr19:15298066 | C | T | exonic | *NOTCH3*:NM_000435exon11:c.1690G>A:p.A564T |
| 265 | Rare | chr19:15298083 | chr19:15298083 | C | T | exonic | *NOTCH3*:NM_000435:exon11:c.1673G>A:p.R558H |
| 266 | Rare | chr19:15298126 | chr19:15298126 | G | A | exonic | *NOTCH3*:NM_000435:exon11:c.1630C>T:p.R544C |
| 267 | Common | chr19:15298136 | chr19:15298136 | C | A | exonic | *NOTCH3*:NM_000435:exon11:c.1620G>T:p.T540T |
| 268 | Rare | chr19:15298175 | chr19:15298175 | C | T | intronic |  |
| 269 | Rare | chr19:15298261 | chr19:15298261 | C | T | intronic |  |
| 270 | Rare | chr19:15298268 | chr19:15298268 | C | T | intronic |  |
| 271 | Common | chr19:15298270 | chr19:15298270 | A | G | intronic |  |
| 272 | Common | chr19:15298509 | chr19:15298509 | C | T | intronic |  |
| 273 | Rare | chr19:15298590 | chr19:15298590 | G | A | intronic |  |
| 274 | Rare | chr19:15298679 | chr19:15298679 | T | G | intronic |  |
| 275 | Rare | chr19:15298720 | chr19:15298720 | G | C | exonic | *NOTCH3*:NM_000435:exon10:c.1578C>G:p.P526P |
| 276 | Rare | chr19:15298790 | chr19:15298790 | G | A | exonic | *NOTCH3*:NM_000435:exon10:c.1508C>T:p.T503M |
| 277 | Rare | chr19:15299048 | chr19:15299048 | G | A | exonic | *NOTCH3*:NM_000435:exon9:c.1490C>T:p.S497L |
| 278 | Rare | chr19:15299085 | chr19:15299085 | T | C | exonic | *NOTCH3*:NM_000435:exon9:c.1453A>G:p.K485E |
| 279 | Rare | chr19:15299143 | chr19:15299143 | A | G | exonic | *NOTCH3*:NM_000435:exon9:c.1395T>C:p.Y465Y |
| 280 | Rare | chr19:15299185 | chr19:15299185 | A | C | intronic |  |
| 281 | Rare | chr19:15299232 | chr19:15299232 | C | G | intronic |  |
| 282 | Rare | chr19:15299252 | chr19:15299252 | C | T | intronic |  |
| 283 | Rare | chr19:15299328 | chr19:15299328 | C | G | intronic |  |
| 284 | Rare | chr19:15299344 | chr19:15299344 | A | G | intronic |  |
| 285 | Common | chr19:15299648 | chr19:15299648 | T | C | intronic |  |
| 286 | Rare | chr19:15299913 | chr19:15299913 | C | A | exonic | *NOTCH3*:NM_000435:exon8:c.1265G>T:p.G422V |
| 287 | Common | chr19:15300069 | chr19:15300069 | T | C | intronic |  |
| 288 | Rare | chr19:15300070 | chr19:15300070 | G | A | intronic |  |
| 289 | Rare | chr19:15300090 | chr19:15300090 | A | C | exonic | *NOTCH3*:NM_000435:exon7:c.1186T>G:p.S396A |
| 290 | Rare | chr19:15300124 | chr19:15300124 | C | A | exonic | *NOTCH3*:NM_000435:exon7:c.1152G>T:p.T384T |
| 291 | Rare | chr19:15300205 | chr19:15300205 | G | A | exonic | *NOTCH3*:NM_000435exon7:c.1071C>T:p.S357S |
| 292 | rare | chr19:15300371 | chr19:15300371 | C | T | intronic |  |
| 293 | Rare | chr19:15300374 | chr19:15300374 | A | T | intronic |  |
| 294 | Rare | chr19:15300384 | chr19:15300384 | C | T | intronic |  |
| 295 | Rare | chr19:15300387 | chr19:15300387 | T | C | intronic |  |
| 296 | Rare | chr19:15300424 | chr19:15300424 | G | A | intronic |  |
| 297 | Rare | chr19:15302036 | chr19:15302036 | G | A | intronic |  |
| 298 | Rare | chr19:15302047 | chr19:15302047 | A | G | intronic |  |
| 299 | Rare | chr19:15302086 | chr19:15302086 | C | G | intronic |  |
| 300 | Rare | chr19:15302090 | chr19:15302090 | C | T | intronic |  |
| 301 | Rare | chr19:15302170 | chr19:15302170 | C | T | intronic |  |
| 302 | Rare | chr19:15302225 | chr19:15302225 | G | A | intronic |  |
| 303 | Rare | chr19:15302341 | chr19:15302341 | G | A | exonic | *NOTCH3*:NM_000435:exon6:c.930C>T:p.S310S |
| 304 | Rare | chr19:15302649 | chr19:15302649 | C | T | exonic | *NOTCH3*:NM_000435:exon5:c.709G>A:p.V237M |
| 305 | Common | chr19:15302844 | chr19:15302844 | T | C | exonic | *NOTCH3*:NM_000435:exon4:c.606A>G:p.A202A |
| 306 | Rare | chr19:15302848 | chr19:15302848 | C | A | exonic | *NOTCH3*:NM_000435:exon4:c.602G>T:p.C201F |
| 307 | Rare | chr19:15302848 | chr19:15302848 | C | G | exonic | *NOTCH3*:NM_000435:exon4:c.602G>C:p.C201S |
| 308 | Rare | chr19:15302860 | chr19:15302860 | G | A | exonic | *NOTCH3*:NM_000435:exon4:c.590C>T:p.P197L |
| 309 | Rare | chr19:15302906 | chr19:15302906 | G | A | exonic | *NOTCH3*:NM_000435:exon4:c.544C>T:p.R182C |
| 310 | Rare | chr19:15302935 | chr19:15302935 | C | T | exonic | *NOTCH3*:NM_000435:exon4:c.515G>A:p.G172D |
| 311 | Rare | chr19:15302944 | chr19:15302944 | C | T | exonic | *NOTCH3*:NM_000435:exon4:c.506G>A:p.R169H |
| 312 | Rare | chr19:15302951 | chr19:15302951 | G | A | exonic | *NOTCH3*:NM_000435:exon4:c.499C>T:p.P167S |
| 313 | Rare | chr19:15302968 | chr19:15302968 | T | C | exonic | *NOTCH3*:NM_000435:exon4:c.482A>G:p.E161G |
| 314 | Rare | chr19:15302979 | chr19:15302979 | G | A | exonic | *NOTCH3*:NM_000435:exon4:c.471C>T:p.S157S |
| 315 | Rare | chr19:15303035 | chr19:15303035 | C | T | exonic | *NOTCH3*:NM_000435:exon4:c.415G>A:p.D139N |
| 316 | Rare | chr19:15303059 | chr19:15303059 | C | G | exonic | *NOTCH3*:NM_000435:exon4:c.391G>C:p.G131R |
| 317 | Rare | chr19:15303059 | chr19:15303059 | C | T | exonic | *NOTCH3*:NM_000435exon4:c.391G>A:p.G131S |
| 318 | Rare | chr19:15303060 | chr19:15303060 | G | A | exonic | *NOTCH3*:NM_000435:exon4:c.390C>T:p.H130H |
| 319 | Rare | chr19:15303077 | chr19:15303077 | T | G | exonic | *NOTCH3*:NM_000435:exon4:c.373A>C:p.S125R |
| 320 | Common | chr19:15303225 | chr19:15303225 | G | A | exonic | *NOTCH3*:NM_000435:exon3:c.303C>T:p.T101T |
| 321 | Rare | chr19:15303259 | chr19:15303259 | C | T | exonic | *NOTCH3*:NM_000435:exon3:c.269G>A:p.R90H |
| 322 | Rare | chr19:15303304 | chr19:15303304 | C | T | exonic | *NOTCH3*:NM_000435:exon3:c.224G>A:p.R75Q |
| 323 | Rare | chr19:15303381 | chr19:15303381 | A | G | intronic |  |
| 324 | Rare | chr19:15303414 | chr19:15303414 | C | T | intronic |  |
| 325 | Rare | chr19:15303511 | chr19:15303511 | G | A | intronic |  |
| 326 | Rare | chr19:15303524 | chr19:15303524 | A | G | intronic |  |
| 327 | rare | chr19:15303532 | chr19:15303532 | C | G | intronic |  |
| 328 | Rare | chr19:15308246 | chr19:15308246 | C | T | intronic |  |
| 329 | Rare | chr19:15308254 | chr19:15308254 | C | A | intronic |  |
| 330 | Rare | chr19:15308263 | chr19:15308263 | C | A | intronic |  |
| 331 | Rare | chr19:15308264 | chr19:15308264 | C | T | intronic |  |
| 332 | Rare | chr19:15308264 | chr19:15308264 | C | A | intronic |  |
| 333 | Rare | chr19:15308265 | chr19:15308265 | G | A | intronic |  |
| 334 | Rare | chr19:15308287 | chr19:15308287 | G | C | intronic |  |
| 335 | Common | chr19:15308490 | chr19:15308490 | T | C | intronic |  |
| 336 | Rare | chr19:15308525 | chr19:15308525 | C | T | intronic |  |
| 337 | Rare | chr19:15308542 | chr19:15308542 | C | A | intronic |  |
| 338 | Rare | chr19:15308546 | chr19:15308546 | C | A | intronic |  |
| 339 | Rare | chr19:15308556 | chr19:15308556 | C | A | intronic |  |
| 340 | Rare | chr19:15311424 | chr19:15311424 | C | T | intronic |  |
| 341 | rare | chr19:15311457 | chr19:15311457 | C | A | intronic |  |
| 342 | Rare | chr19:15311462 | chr19:15311462 | A | G | intronic |  |
| 343 | Rare | chr19:15311574 | chr19:15311574 | C | A | intronic |  |
| 344 | Rare | chr19:15311612 | chr19:15311612 | C | T | exonic | *NOTCH3*:NM_000435:exon1:c.105G>A:p.G35G |
| 345 | Rare | chr19:15311621 | chr19:15311621 | C | G | exonic | *NOTCH3*:NM_000435:exon1:c.96G>C:p.L32L |

†, variant identified by complementary Sanger sequencing; ‡, variant found by both target sequencing and complementary Sanger sequencing.

Abbreviations: AD, Alzheimer's disease; SVaD, subcortical vascular dementia; Chr, [chromosome](javascript:;); Ref, reference allele; Alt, altered allele; UTR3, untranslated region 3;

**Supplementary Table S2.** **Cysteine-sparing *NOTCH3* mutations in SVaD patients without** **cysteine-altering *NOTCH3* variant and controls.**

| **Position** | **Nucleotide change** | **amino acid change** | **Exon** | **SVaD without cysteine-altering *NOTCH3* variant (n=80)** | **Control(n=365)** |
| --- | --- | --- | --- | --- | --- |
| Chr19: 15271796 | c.6643C>G | p.P2215A | 33 | 0 | 1 Het |
| Chr19: 15272005 | c.6434G>A | p.R2145Q | 33 | 1 Het | 0 |
| Chr19: 15273335 | c.5854G>A | p.V1952M | 32 | 0 | 1 Het |
| Chr19: 15276193 | c.5801C>T | p.A1934V | 31 | 0 | 1 Het |
| Chr19: 15276230 | c.5764G>T | p.V1922L | 30 | 1 Het | 0 |
| Chr19: 15276756 | c.5509C>T | p.R1837C | 30 | 0 | 1 Het |
| Chr19: 15281175 | c.5081G>A | p.R1694Q | 27 | 0 | 1 Het |
| Chr19: 15281582 | c.4791T>A | p.N1597K | 26 | 0 | 2 Het |
| Chr19: 15288391 | c.4348G>A | p.A1450T | 24 | 0 | 1 Het |
| Chr19: 15288700 | c.4039G>C | p.G1347R | 24 | 1 Het | 1 Het |
| Chr19: 15289994 | c.3560G>A | p.G1187D | 22 | 0 | 1 Het |
| Chr19: 15290030 | c.3524G>A | p.R1175Q | 22 | 0 | 1 Het |
| Chr19: 15290911 | c.3299G>A | p.R1100H | 20 | 1 Het | 1 Het |
| Chr19: 15291860 | c.2906G>A | p.R969Q | 14 | 0 | 1 Het |
| Chr19: 15298066 | c.1690G>A | p.A564T | 11 | 1 Het | 0 |
| Chr19: 15298083 | c.1673G>A | p.R558H | 11 | 0 | 1 Het |
| Chr19: 15299048 | c.1490C>T | p.S497L | 9 | 0 | 1 Het |
| Chr19: 15302649 | c.709G>A | p.V237M | 5 | 0 | 2 Het |
| Chr19: 15302935 | c.515G>A | p.G172D | 4 | 0 | 1 Het |
| Chr19: 15302944 | c.506G>A | p.R169H | 4 | 0 | 1 Het |
| Chr19: 15302951 | c.499C>T | p.P167S | 4 | 0 | 4 Het |
| Chr19: 15302968 | c.482A>G | p.E161G | 4 | 0 | 1 Het |
| Chr19: 15303059 | c.391G>C | p.G131R | 4 | 0 | 1 Het |
| Chr19: 15303059 | c.391G>A | p.G131S | 4 | 1 Het | 0 |
| Chr19: 15303304 | c.224G>A | p.R75Q | 3 | 0 | 1 Het |

Abbreviations: Chr, [chromosome](javascript:;); SVaD, subcortical vascular dementia; Het, heterozygote.

**Supplementary Table S3. Association analysis of *NOTCH3* common variants in AD cases and healthy elderly controls.**

| **Position** | **Rs number** | **MA** | **MA** | | ***p*** | ***p_adj_*** |
| --- | --- | --- | --- | --- | --- | --- |
|  |  |  | **AD(n=676)** | **Control(n=365)** |  |  |
| Chr19:15270536 | rs16980378 | G | 0.037 | 0.029 | 0.339 | 0.768 |
| Chr19:15270583 | rs1044123 | G | 0.450 | 0.438 | 0.618 | 0.336 |
| Chr19:15270636 | rs12082 | T | 0.130 | 0.110 | 0.168 | 0.060 |
| Chr19:15270665 | rs1044116 | C | 0.450 | 0.438 | 0.618 | 0.336 |
| Chr19:15270805 | rs1044055 | C | 0.451 | 0.438 | 0.595 | 0.317 |
| Chr19:15271771 | rs1044009 | G | 0.448 | 0.434 | 0.540 | 0.286 |
| Chr19:15272001 | rs1044008 | T | 0.002 | 0.005 | 0.252 | 0.235 |
| Chr19:15273221 | rs757472 | T | 0.194 | 0.200 | 0.749 | 0.945 |
| Chr19:15273231 | rs4809028 | A | 0.194 | 0.200 | 0.749 | 0.945 |
| Chr19:15273248 | rs4809029 | A | 0.194 | 0.200 | 0.749 | 0.945 |
| Chr19:15273381 | rs4809030 | A | 0.194 | 0.200 | 0.749 | 0.945 |
| Chr19:15276007 | [rs2886698](https://www.ncbi.nlm.nih.gov/SNP/snp_ref.cgi?rs=2886698) | C | 0.448 | 0.433 | 0.522 | 0.251 |
| Chr19:15276055 | [rs2074617](https://www.ncbi.nlm.nih.gov/SNP/snp_ref.cgi?rs=2074617) | C | 0.196 | 0.201 | 0.755 | 0.958 |
| Chr19:15276143 | rs2074618 | C | 0.196 | 0.201 | 0.755 | 0.958 |
| Chr19:15276919 | rs2074619 | G | 0.196 | 0.201 | 0.755 | 0.958 |
| Chr19:15276923 | rs10416777 | C | 0.196 | 0.201 | 0.755 | 0.958 |
| Chr19:15277006 | rs45591935 | T | 0.196 | 0.201 | 0.755 | 0.958 |
| Chr19:15277009 | rs62113791 | C | 0.196 | 0.201 | 0.755 | 0.958 |
| Chr19:15277010 | rs45504393 | A | 0.196 | 0.201 | 0.755 | 0.958 |
| Chr19:15277027 | [rs10415431](https://www.ncbi.nlm.nih.gov/SNP/snp_ref.cgi?rs=10415431) | G | 0.194 | 0.201 | 0.693 | 0.994 |
| Chr19:15277938 | [rs1548554](https://www.ncbi.nlm.nih.gov/SNP/snp_ref.cgi?rs=1548554) | C | 0.194 | 0.200 | 0.750 | 0.945 |
| Chr19:15278057 | rs1548555 | A | 0.194 | 0.200 | 0.750 | 0.945 |
| Chr19: 15278335 | rs552713969 | T | 0.001 | 0.004 | 0.130 | 0.102 |
| Chr19:15280765 | [rs7249115](https://www.ncbi.nlm.nih.gov/SNP/snp_ref.cgi?rs=7249115) | A | 0.196 | 0.203 | 0.730 | 0.976 |
| Chr19:15281386 | rs2074620 | C | 0.451 | 0.438 | 0.595 | 0.317 |
| Chr19: 15281459 | rs56277836 | G | 0.001 | 0.001 | 1.000 | 0.634 |
| Chr19:15285052 | rs1044006 | T | 0.195 | 0.200 | 0.781 | 0.923 |
| Chr19: 15285063 | rs141320511 | T | 0.06 | 0.007 | 0.779 | 0.855 |
| Chr19:15285272 | rs10422818 | T | 0.196 | 0.200 | 0.844 | 0.888 |
| Chr19:15285422 | [rs187775089](https://www.ncbi.nlm.nih.gov/SNP/snp_ref.cgi?rs=187775089) | A | 0.023 | 0.018 | 0.414 | 0.334 |
| Chr19:15289613 | rs11670823 | A | 0.463 | 0.452 | 0.625 | 0.417 |
| Chr19:15289763 | rs78907190 | A | 0.010 | 0.011 | 0.793 | 0.901 |
| Chr19:15290031 | rs200504060 | A | 0.006 | 0.005 | 1.000 | 0.820 |
| Chr19:15290125 | rs56061231 | G | 0.463 | 0.452 | 0.648 | 0.421 |
| Chr19:15290412 | rs2074621 | G | 0.462 | 0.451 | 0.629 | 0.400 |
| Chr19:15291096 | rs137943075 | A | 0.060 | 0.058 | 0.770 | 0.864 |
| Chr19:15291136 | rs60360024 | A | 0.094 | 0.084 | 0.410 | 0.302 |
| Chr19:15291576 | rs35769976 | G | 0.000 | 0.001 | 0.354 | 1.000 |
| Chr19:15291699 | rs57538005 | C | 0.000 | 0.001 | 0.354 | 1.000 |
| Chr19:15292088 | rs7257550 | G | 0.177 | 0.175 | 0.929 | 0.708 |
| Chr19:15292271 | rs11669950 | C | 0.463 | 0.449 | 0543 | 0.315 |
| Chr19:15292365 | rs568244512 | A | 0.007 | 0.010 | 0.731 | 0.880 |
| Chr19:15292366 | rs11669982 | C | 0.178 | 0.175 | 0.895 | 0.667 |
| Chr19:15292437 | rs1043997 | T | 0.178 | 0.175 | 0.862 | 0.643 |
| chr19:15292599 | rs201436750 | A | 0.001 | 0.001 | 1.000 | 0.468 |
| Chr19:15294991 | rs2074616 | C | 0.138 | 0.140 | 0.910 | 0.895 |
| Chr19:15295134 | rs1043996 | G | 0.421 | 0.415 | 0.784 | 0.542 |
| Chr19:15297576 | rs7245563 | T | 0.288 | 0.278 | 0.638 | 0.510 |
| Chr19:15298136 | rs75617410 | A | 0.044 | 0.049 | 0.598 | 0.859 |
| Chr19:15299648 | rs2238643 | C | 0.489 | 0.507 | 0.432 | 0.421 |
| Chr19:15300069 | rs10423702 | T | 0.133 | 0.130 | 0.833 | 0.694 |
| Chr19:15302844 | rs1043994 | T | 0.133 | 0.132 | 0.902 | 0.765 |
| Chr19:15303225 | rs3815188 | A | 0.375 | 0.353 | 0.335 | 0.371 |

Abbreviations: AD, Alzheimer's disease; Chr, [chromosome](javascript:;); MA, minor allele; MAF, minor allele frequency; *p*_adj_, *p*-value after the adjustment of age, gender, and APOE ε4 status.

**Supplementary Table S4. Rare missense mutations in *NOTCH3* included in the gene-based association analysis**.

| **Position** | **Nucleotide change** | **amino acid change** | **Exon** | **AD(n=676)** | **Control(n=365)** |
| --- | --- | --- | --- | --- | --- |
| Chr19: 15271654 | c.6785C>T | p.S2262F | 33 | 1 Het | 0 |
| Chr19: 15271796 | c.6643C>G | p.P2215A | 33 | 0 | 1 Het |
| Chr19: 15271813 | c.6626C>T | p.P2209L | 33 | 1 Het | 0 |
| Chr19: 15271819 | c.6620G>A | p.R2207Q | 33 | 1 Het | 0 |
| Chr19: 15271820 | c.6619C>T | p.R2207W | 33 | 1 Het | 0 |
| Chr19: 15271867 | c.6572C>T | p.P2191L | 33 | 1 Het | 0 |
| Chr19: 15272239 | c.6200C>T | p.P2067L | 33 | 1 Het | 0 |
| Chr19: 15272339 | c.6100C>T | p.P2034S | 33 | 1 Het | 0 |
| Chr19: 15273335 | c.5854G>A | p.V1952M | 32 | 0 | 1 Het |
| Chr19: 15276193 | c.5801C>T | p.A1934V | 31 | 1 Het | 1 Het |
| Chr19: 15276717 | c.5548G>A | p.A1850T | 30 | 2 Het | 0 |
| Chr19: 15276755 | c.5510G>A | p.R1837H | 30 | 2 Het | 0 |
| Chr19: 15276756 | c.5509C>T | p.R1837C | 30 | 0 | 1 Het |
| Chr19: 15276863 | c.5402G>A | p.G1801E | 30 | 1 Het | 0 |
| Chr19: 15278069 | c.5353C>T | p.R1785C | 29 | 1 Het | 0 |
| Chr19: 15280910 | c.5186C>T | p.A1729V | 28 | 1 Het | 0 |
| Chr19: 15281175 | c.5081G>A | p.R1694Q | 27 | 0 | 1 Het |
| Chr19: 15281185 | c.5071A>G | p.K1691E | 27 | 1 Het | 0 |
| Chr19: 15281281 | c.4975G>A | p.V1659I | 27 | 1 Het | 0 |
| Chr19: 15281580 | c.4793A>T | p.D1598V | 26 | 1 Het | 0 |
| Chr19: 15281582 | c.4791T>A | p.N1597K | 26 | 2 Het | 2 Het |
| Chr19: 15284909 | c.4706C>T | p.A1569V | 25 | 1 Het | 0 |
| Chr19: 15288391 | c.4348G>A | p.A1450T | 24 | 3 Het | 1 Het |
| Chr19: 15288568 | c.4171G>C | p.A1391P | 24 | 1 Het | 0 |
| Chr19: 15288678 | c.4061C>T | p.P1354L | 24 | 1 Het | 0 |
| Chr19: 15288700 | c.4039G>C | p.G1347R | 24 | 11 Het | 1 Het |
| Chr19: 15288793 | c.3946C>T | p.P1316S | 24 | 1 Het | 0 |
| Chr19: 15289994 | c.3560G>A | p.G1187D | 22 | 0 | 1 Het |
| Chr19: 15290030 | c.3524G>A | p.R1175Q | 22 | 0 | 1 Het |
| Chr19: 15290180 | c.3455C>T | p.T1152M | 21 | 1 Het | 0 |
| Chr19: 15290911 | c.3299G>A | p.R1100H | 20 | 1 Het | 1 Het |
| Chr19: 15290950 | c.3260C>T | p.P1087L | 20 | 1 Het | 0 |
| Chr19: 15291783 | c.2983C>T | p.P995S | 18 | 1 Het | 0 |
| Chr19: 15291860 | c.2906G>A | p.R969Q | 14 | 0 | 1 Het |
| Chr19: 15296130 | c.2234C>T | p.A745V | 12 | 1 Het | 0 |
| Chr19: 15297978 | c.1778A>G | p.H593R | 11 | 1 Het | 0 |
| Chr19: 15298083 | c.1673G>A | p.R558H | 11 | 0 | 1 Het |
| Chr19: 15298790 | c.1508C>T | p.T503M | 10 | 1 Het | 0 |
| Chr19: 15299048 | c.1490C>T | p.S497L | 9 | 1 Het | 1 Het |
| Chr19: 15299085 | c.1453A>G | p.K485E | 9 | 1 Het | 0 |
| Chr19: 15299913 | c.1265G>T | p.G422V | 8 | 1 Het | 0 |
| Chr19: 15300090 | c.1186T>G | p.S396A | 7 | 1 Het | 0 |
| Chr19: 15302649 | c.709G>A | p.V237M | 5 | 5 Het | 2 Het |
| Chr19: 15302860 | c.590C>T | p.P197L | 4 | 1 Het | 0 |
| Chr19: 15302935 | c.515G>A | p.G172D | 4 | 1 Het | 1 Het |
| Chr19: 15302944 | c.506G>A | p.R169H | 4 | 0 | 1 Het |
| Chr19: 15302951 | c.499C>T | p.P167S | 4 | 7 Het | 4 Het |
| Chr19: 15302968 | c.482A>G | p.E161G | 4 | 0 | 1 Het |
| Chr19: 15303035 | c.415G>A | p.D139N | 4 | 1 Het | 0 |
| Chr19: 15303059 | c.391G>C | p.G131R | 4 | 0 | 1 Het |
| Chr19: 15303077 | c.373A>C | p.S125R | 4 | 1 Het | 0 |
| Chr19: 15303259 | c.269G>A | p.R90H | 3 | 2 Het | 0 |
| Chr19: 15303304 | c.224G>A | p.R75Q | 3 | 1 Het | 1 Het |

Abbreviations: Chr, [chromosome](javascript:;); AD, Alzheimer's disease; Het, heterozygote.

**Figure legends**

**Supplementary Figure S1.** Brain MRI and electron microscopy pictures of the patient with the variant c.4039G>C,p.G1347R. Axial brain MRI imaging of an AD patient carried the *NOTCH3* p.G1347R (c.403 9G>C) mutation showed moderate paraventricular white matter hyperintensities on FLAIR (A-C) and numerous microbleeds on SWI (D). Skin biopsy showed no GOM located on the cell membrane of the vascular smooth muscle cell, ×2,500(E).


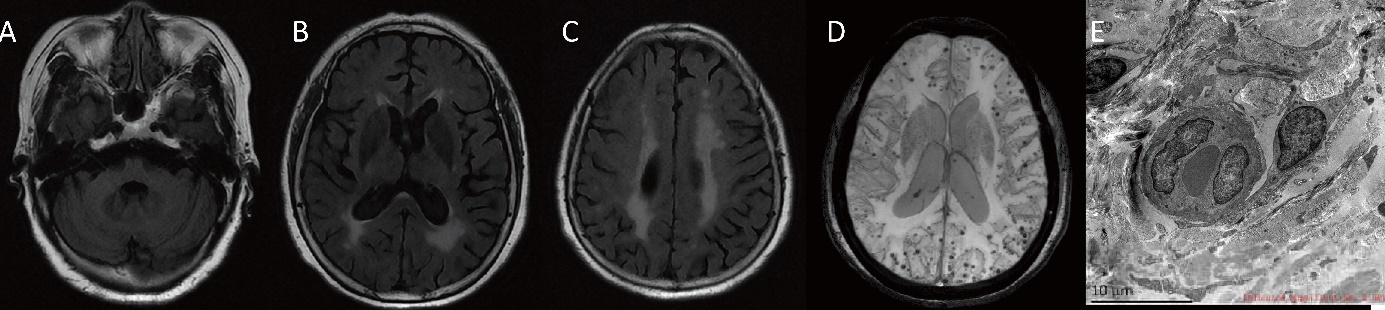

Supplement: Supplementary file 1 — Supplementary Material [file CNS-27-930-s001.docx]
